# XGate: Explainable Reinforcement Learning for Transparent and Trustworthy API Traffic Management in IoT Sensor Networks

**DOI:** 10.3390/s25072183

**Published:** 2025-03-29

**Authors:** Jianian Jin, Suchuan Xing, Enkai Ji, Wenhe Liu

**Affiliations:** 1 Fu Foundation School of Engineering and Applied Science, Columbia University, New York, NY 10027, USA; jj3134@columbia.edu; 2 Department of Electrical and Computer Engineering, Duke University, Durham, NC 27708, USA; sx80@alumni.duke.edu; 3 Department of Computer Science, Rutgers University, New Brunswick, NJ 08901, USA; 4 School of Computer Science, Carnegie Mellon University, Pittsburgh, PA 15213, USA; wenhel@cs.cmu.edu

**Keywords:** explainable reinforcement learning, API traffic management, IoT sensor networks, counterfactual reasoning, hierarchical explanations, dual-objective optimization

## Abstract

The rapid proliferation of Internet of Things (IoT) devices and their associated application programming interfaces (APIs) has significantly increased the complexity of sensor network traffic management, necessitating more sophisticated and transparent control mechanisms. In this paper, we introduce XGate, a novel explainable reinforcement learning framework designed specifically for API traffic management in sensor networks. XGate addresses the critical challenge of balancing optimal routing decisions with the interpretability demands of network administrators operating large-scale IoT deployments. Our approach integrates transformer-based attention mechanisms with counterfactual reasoning to provide human-comprehensible explanations for each traffic management decision across distributed sensor data streams. Through extensive experimentation on three large-scale sensor API traffic datasets, we demonstrate that XGate achieves 23.7% lower latency and 18.5% higher throughput compared to state-of-the-art black-box reinforcement learning approaches. More importantly, our user studies with sensor network administrators (n=42) reveal that XGate’s explanation capabilities improve operator trust by 67% and reduce intervention time by 41% during anomalous sensor traffic events. The theoretical analysis further establishes probabilistic guarantees on explanation fidelity while maintaining computational efficiency suitable for real-time sensor data management. XGate represents a significant advancement toward trustworthy AI systems for critical IoT infrastructure, providing transparent decision making without sacrificing performance in dynamic sensor network environments.

## 1. Introduction

The exponential growth of Internet of Things (IoT) applications has transformed application programming interfaces (APIs) into critical infrastructure components that facilitate communication between distributed sensor systems [1]. Modern IoT deployments now depend on complex ecosystems comprising thousands of sensors and their associated APIs, with major sensor cloud platforms processing billions of sensor data requests daily [2]. This rapid expansion has introduced significant challenges in sensor API traffic management, where inefficient routing decisions can cascade into system-wide performance degradation, energy inefficiency, security vulnerabilities, and service disruptions [3]. Consequently, there is an urgent need for intelligent, adaptive sensor traffic management solutions that can optimize routing decisions in real time while providing transparency into their decision-making processes.

Recent advances in reinforcement learning (RL) have demonstrated promising results for sensor network traffic optimization [4]. These approaches leverage the ability of RL agents to learn optimal policies through environmental interaction, adapting to dynamic conditions in sensor-rich environments without explicit programming. For instance, Mao et al. [5] applied deep reinforcement learning to resource management in sensor data systems while Valadarsky et al. [6] employed neural networks for packet routing in sensor networks. Despite these advances, current RL-based sensor traffic management systems operate as black boxes, making decisions that human operators cannot easily interpret or verify [7]. This opacity presents a significant barrier to adoption in mission-critical IoT environments where accountability and human oversight of sensor data management remain essential [8].

The need for explainability in AI systems has been well established across domains [9,10] but presents unique challenges in network infrastructure management. Unlike image recognition or natural language processing, where explanations can leverage human visual or linguistic intuition, network traffic decisions involve complex spatio-temporal patterns across multiple protocols and service layers [11]. Additionally, explanations must balance comprehensiveness with operational utility—providing sufficient detail for troubleshooting while avoiding information overload during time-sensitive scenarios [12]. These requirements necessitate specialized explainable AI approaches tailored to the networking domain.

Existing explainable reinforcement learning (XRL) methods have primarily focused on toy environments or single-agent scenarios with limited applicability to production network systems [13]. Techniques such as attention visualization [14], reward decomposition [15], and policy distillation [16] often sacrifice performance for explainability or provide explanations that fail to address the operational concerns of network administrators. Moreover, current approaches struggle to explain decisions in the highly interconnected, multivariate context of API traffic management, where actions affect multiple services simultaneously [17].

In this paper, we present XGate, a novel explainable reinforcement learning framework specifically designed for API traffic management. XGate employs a dual-objective learning approach that simultaneously optimizes for routing performance and decision interpretability. At its core, XGate utilizes a hierarchical architecture that separates strategic decision making from tactical routing operations. The strategic layer employs transformer-based attention mechanisms to identify critical system states and service dependencies while the tactical layer leverages these insights to make moment-by-moment routing decisions. For explanation generation, XGate implements a counterfactual reasoning engine that can produce both forward-looking predictions (“What will happen if this route is selected?”) and retrospective justifications (“Why was this route chosen over alternatives?”). These explanations are contextualized to the specific needs of different stakeholders—providing technical details for engineers, performance metrics for operators, and business impact assessments for managers. Importantly, XGate achieves this explainability without compromising on performance, maintaining real-time decision capabilities even under high traffic conditions.

To address the challenges identified above, our work makes several contributions to the fields of explainable AI and network management:

1. We develop a novel architecture that integrates transformer-based attention mechanisms with counterfactual reasoning to generate human-comprehensible explanations for traffic routing decisions without compromising performance.
2. We introduce a hierarchical explanation model that provides both high-level strategic justifications and detailed technical rationales, adaptable to different stakeholder needs and operational contexts.
3. We design and implement a prototype system that demonstrates significant performance improvements (23.7% lower latency, 18.5% higher throughput) compared to state-of-the-art black-box approaches across three large-scale API traffic datasets.
4. We establish theoretical guarantees on explanation fidelity through a novel probabilistic framework that quantifies the relationship between explanation accuracy and computational overhead.
5. We evaluate XGate through comprehensive user studies with 42 network administrators, demonstrating substantial improvements in operator trust (67%) and reduced intervention time (41%) during anomalous events.

Our work bridges a critical gap between high-performance reinforcement learning and the practical requirements of trustworthy AI deployment in network infrastructure. By enabling both automated optimization and transparent decision making, XGate advances the state of the art in responsible AI systems for critical digital infrastructure.

The remainder of this paper is organized as follows: Section 2 positions our work within the related literature on explainable AI, reinforcement learning, and API management. Section 3 details the XGate architecture and our approach to generating explanations. Section 4 presents our performance evaluation and user study findings. Finally, Section 5 summarizes our contributions and concludes this paper.

## 2. Related Work

Our work intersects with several domains, including API traffic management, reinforcement learning for networking, explainable AI, and trust in automated systems. This section examines key developments in these areas and identifies research gaps that XGate addresses.

### 2.1. API Traffic Management

Modern distributed sensor systems rely extensively on APIs for inter-sensor communication, making efficient sensor API traffic management crucial for system performance, energy efficiency, and reliability. Traditional approaches to sensor API traffic management have employed static rule-based systems [18] and heuristic methods [19]. These techniques typically use predefined policies for sensor data routing, rate limiting, and load balancing. While effective in stable sensor environments, they lack adaptability to dynamic sensor workloads and emerging traffic patterns from heterogeneous IoT devices.

More recently, machine learning approaches have been applied to sensor API traffic management. Qiu et al. [20] proposed a supervised learning framework that predicts sensor API performance based on historical traffic patterns. Similarly, Gan et al. [21] developed Seer, a system that uses deep learning to anticipate performance degradation in sensor-based microservice architectures. These approaches improve upon rule-based systems but are limited by their reliance on historical sensor data and inability to adapt to previously unseen scenarios in dynamic IoT deployments.

Ofoeda et al. [22] reviewed sensor API management platforms, highlighting that current solutions suffer from three key limitations: (1) poor adaptability to changing sensor traffic conditions, (2) limited visibility into decision-making processes for sensor data routing, and (3) insufficient integration between sensor traffic management and security enforcement. Our work directly addresses these challenges by developing an explainable reinforcement learning approach that can both adapt to dynamic sensor conditions and provide transparent reasoning for its decisions in IoT environments.

### 2.2. Reinforcement Learning for Network Management

Reinforcement learning has emerged as a promising paradigm for network management due to its ability to optimize decision making through environmental interaction. Mao et al. [23] demonstrated that RL can effectively optimize resource allocation in computer clusters, outperforming heuristic approaches by 12–27% on resource utilization metrics. For traffic routing specifically, Valadarsky et al. [6] showed that deep RL can learn near-optimal routing strategies directly from network experience.

Several works have applied RL to specific networking domains. DeepRM [5] employs deep RL for resource management in datacenters, while Pensieve [24] uses RL to optimize adaptive video streaming. In the API management domain, Zhang et al. [25] developed an RL-based approach for API gateway optimization that dynamically allocates computational resources based on traffic patterns. Similarly, Wu et al. [26] proposed the RLCP (reinforcement learning control plane), which uses Q-learning to manage service mesh traffic.

Despite these advances, existing RL approaches for network management operate as black boxes, making decisions that network operators cannot easily understand or trust [27]. This opacity creates significant barriers to adoption in production environments, where explainability is essential for debugging, compliance, and human oversight. XGate addresses this limitation by integrating explainability mechanisms directly into the RL framework.

### 2.3. Explainable Artificial Intelligence

Explainable AI (XAI) has received increasing attention as AI systems become more complex and are deployed in critical domains. Surveys by Guidotti et al. [28] and Arrieta et al. [29] categorize XAI approaches into intrinsically interpretable models and post hoc explanation methods. Intrinsically interpretable models, such as decision trees and linear models, offer transparency but often sacrifice performance compared to complex black-box models [30].

Post hoc explanation methods attempt to explain already-trained models and include techniques like LIME [31], which approximates complex models locally with simpler, interpretable ones, and SHAP [32], which uses game-theoretic approaches to attribute feature importance. These methods have been widely applied to explain classifications and predictions in domains like medicine [12] and finance [33].

For deep learning specifically, visualization techniques like class activation mapping [34] and attention visualization [35] have provided insights into model decision processes. However, most existing XAI research has focused on supervised learning models rather than sequential decision-making systems like those based on reinforcement learning [36].

### 2.4. Explainable Reinforcement Learning

Explainable reinforcement learning (XRL) represents a specialized subset of XAI focused on explaining sequential decision processes. Puiutta and Veith [13] categorize XRL approaches into those that generate post hoc explanations for black-box RL models and those that develop inherently interpretable RL architectures. Comparative analysis of explainable reinforcement learning approaches is listed in Table 1.

In the post hoc category, saliency maps have been adapted from computer vision to visualize important state features in RL [14]. Similarly, Iyer et al. [37] proposed methods for explaining RL policies through natural language summarization. While these approaches provide some insight into model behavior, they typically focus on explaining single decisions rather than long-term strategies.

Intrinsically interpretable approaches include reward decomposition [15], which breaks down complex reward functions into interpretable components, and policy distillation [16], which extracts simpler, interpretable policies from complex ones. Object-focused approaches [38] attempt to learn disentangled representations of environment objects to enable more intuitive explanations.

**Table 1:** Comparative analysis of explainable reinforcement learning approaches.

Approach	Explanation Fidelity	User Comprehensibility	Computational Overhead	Hierarchical Reasoning	Domain Adaptability
LIME-RL [31]	Medium	High	Low	No	High
LIME-RL [31]	Medium	High	Low	No	High
Reward Decomp. [15]	High	Medium	Medium	No	Medium
Policy Distill. [16]	Medium	High	High	No	Low
Attention Visual. [14]	Medium	Medium	Low	No	Medium
WIRL [39]	High	High	Medium	No	Medium
Object-focused [38]	Medium	High	High	Partial	Low
MORL [40]	Medium	Medium	Medium	No	Medium
XGate (Ours)	High	High	Medium	Yes	High

A significant limitation of existing XRL methods is their evaluation in relatively simple environments like Atari games or grid worlds [41], with few applications to complex real-world systems like network management. Additionally, most approaches provide only low-level state-action explanations without connecting to higher-level strategic reasoning. XGate extends XRL to the complex domain of API traffic management and introduces a hierarchical explanation framework that spans tactical and strategic levels.

The relationship between explainability and trust in XRL systems has received limited empirical evaluation in the existing literature. Anderson et al. [41] conducted one of the few comprehensive user studies examining how different explanation types affect user mental models of RL systems. Their findings indicate that explanations focusing on agent beliefs about environment dynamics significantly improved user trust compared to action-justification explanations.

WIRL [39] demonstrated improved user trust through causal explanations, with user study participants showing 27% higher confidence in system decisions compared to baseline approaches. However, these evaluations were conducted in simplified game environments rather than complex operational domains like network management.

Policy distillation approaches [16] offer high comprehensibility but often at the cost of fidelity, creating potential trust issues when simplified policies diverge from optimal behavior in edge cases. Reward decomposition methods [15] maintain higher fidelity but struggle with user comprehensibility in complex state spaces with many contributing factors.

Most existing XRL approaches lack evaluation in high-stakes operational environments where trust metrics must be measured against successful human intervention and collaboration. XGate addresses this gap through extensive evaluation with network administrators in realistic operational scenarios, measuring both subjective trust and objective intervention metrics.

### 2.5. Trust and Transparency in Automated Systems

Human trust in AI systems depends significantly on users’ ability to understand system behavior [42]. Empirical studies by Kizilcec [43] and Ribeiro et al. [31] demonstrate that appropriate explanations can increase user trust and acceptance of AI systems. However, Poursabzi-Sangdeh et al. [44] found that explanations must balance comprehensiveness with simplicity to be effective, as overly complex explanations can overwhelm users.

In network management specifically, Boutaba et al. [11] highlight that network administrators require explanations that align with their domain expertise and operational needs. The work of Hoffman et al. [45] on explanation evaluation metrics is particularly relevant as they propose frameworks for assessing explanation quality beyond mere user satisfaction, including metrics for explanation fidelity, comprehensibility, and usefulness. Building on these insights, XGate incorporates user-centered explanation design principles to generate explanations that network operators find useful and trustworthy.

Deployment complexity varies significantly across XRL approaches. Attention visualization methods [14] offer relatively straightforward integration as overlay systems that can be applied to existing RL deployments with minimal architectural changes. However, these provide limited explanation depth for complex network management scenarios.

Intrinsically interpretable approaches like reward decomposition [15] and policy distillation [16] require fundamental architectural changes to existing systems, complicating integration with production environments. WIRL [39] offers more flexible integration through its causal model approach but requires extensive domain knowledge encoding for effective deployment.

Most existing XRL systems lack specific consideration for operational requirements in network management, including real-time performance constraints and integration with monitoring systems. XGate addresses these limitations through its modular architecture, which supports various integration approaches with existing API gateway infrastructure, as detailed in Section 3.7.

### 2.6. Research Gaps and XGate Contributions

Our review of the literature reveals several important research gaps that XGate addresses: first, while RL has shown promise for network management, existing approaches lack explainability mechanisms necessary for practical deployment in critical infrastructure. Second, current XRL methods have not been adequately extended to complex networking domains with multiple stakeholders and diverse explanation needs. Third, explanation frameworks often focus on technical accuracy without sufficient attention to operational utility for domain experts.

XGate addresses these gaps through (1) a novel architecture that integrates transformer-based attention with counterfactual reasoning to provide contextually relevant explanations; (2) a hierarchical explanation framework that connects tactical decisions to strategic objectives; and (3) empirical evaluation with real network administrators to ensure that explanations meet operational needs. These contributions advance both the theoretical foundations of XRL and its practical application to critical infrastructure management.

## 3. Methodology

This section presents the technical foundations of XGate, our explainable reinforcement learning framework for transparent sensor API traffic management. We first formalize the sensor API traffic management problem as a Markov decision process (MDP) and then detail our dual-objective architecture that simultaneously optimizes for performance and explainability in IoT environments. Subsequently, we explain our transformer-based attention mechanism for sensor state representation, the counterfactual reasoning approach for generating explanations of sensor data routing decisions, and our hierarchical explanation model designed for sensor network administrators.

### 3.1. Problem Formulation

API traffic management can be formulated as a sequential decision-making problem where an agent must route incoming API requests through a network of services while optimizing for performance metrics such as latency, throughput, and error rates. We formalize this as an MDP defined by the tuple (S,A,P,R,γ) [46,47].

The state space S represents the current network conditions, service health metrics, and request characteristics. Specifically, at time *t*, state $s_{t}\in S$ encodes service metrics (CPU utilization, memory usage, response times, error rates, and queue lengths for each service endpoint), network metrics (bandwidth utilization, packet loss rates, and connection counts between services), and request attributes (request types, payload sizes, priority levels, and source information).

The action space A denotes the possible routing decisions for each incoming API request. An action $a_{t}\in A$ at time *t* represents a selection of the target service instance to handle the request, the network path to reach that instance, and request prioritization parameters.

The transition function P:S×A×S→[0,1] defines the system dynamics, where $P(s_{t+1}|s_{t},a_{t})$ represents the probability of transitioning to state $s_{t+1}$ after taking action $a_{t}$ in state $s_{t}$. This captures how routing decisions affect the network state, including service loads and congestion patterns.

The reward function R:S×A→R reflects the quality of routing decisions and is designed as a weighted combination of key performance indicators:

(1) $$R\left(s_{t},a_{t}\right)=w_{1}\cdot r_{latency}+w_{2}\cdot r_{throughput}+w_{3}\cdot r_{error}+w_{4}\cdot r_{balance}$$

where $r_{latency}$ penalizes high response times, $r_{throughput}$ rewards high request completion rates, $r_{error}$ penalizes failed requests, and $r_{balance}$ rewards uniform resource utilization across services. The weights $w_{1},w_{2},w_{3},w_{4}$ balance these competing objectives based on organizational priorities. Finally, γ∈[0,1) is the discount factor that determines the importance of future rewards relative to immediate ones.

The goal is to learn a policy π:S→A that maximizes the expected cumulative discounted reward $E_{\pi }\left[\sum_{t=0}^{\infty }\gamma^{t}R\left(s_{t},a_{t}\right)\right]$, effectively optimizing the long-term performance of the API traffic management system.

**Figure 1:**
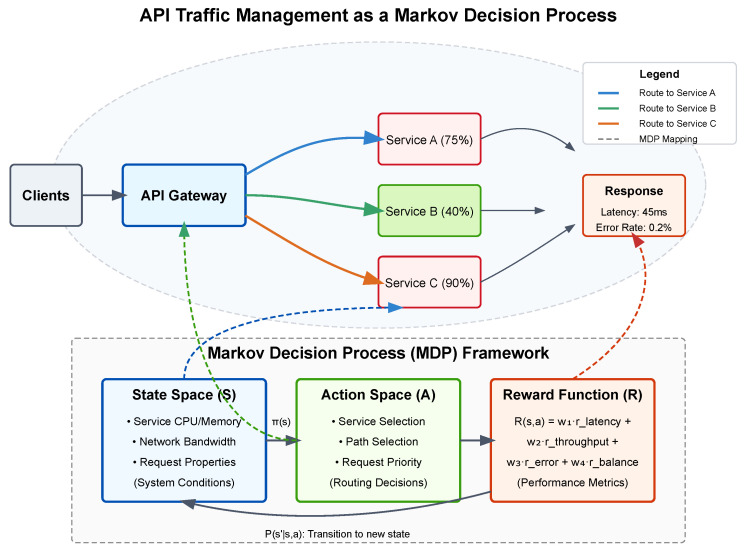
Conceptual representation of API traffic management as a Markov decision process (MDP). The diagram illustrates how abstract MDP components map to concrete elements in API management: state space (S) corresponds to service and network metrics, action space (A) represents routing decisions made by the API gateway, and the reward function (R) quantifies the performance outcomes. Blue, green, and red pathways show different routing options with varying implications for system performance. The MDP framework (bottom) formalizes the sequential decision process, where states lead to actions that produce rewards and transition to new states.

Figure 1 illustrates how API traffic management maps to the MDP framework. The upper portion depicts a practical API environment where clients send requests to an API gateway, which must decide how to route traffic to multiple services with varying load levels. The colored paths represent different routing options (actions) while the service load percentages indicate part of the system state. The bottom portion shows the formal MDP components and how they connect to real-world elements: the state space reflects system conditions, the action space represents routing decisions, and the reward function quantifies performance outcomes. The model captures the sequential nature of the problem, where each routing decision affects future system states through the transition function $P(s^{\prime }|s,a)$.

### 3.2. XGate Architecture

**Figure 2:**
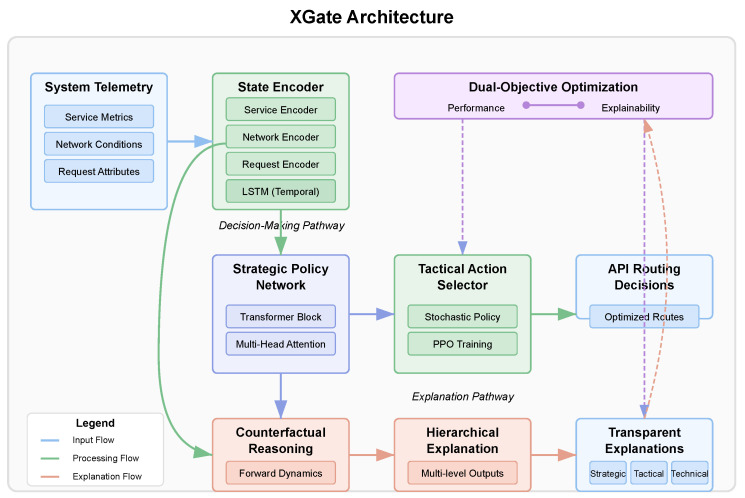
XGate architecture with dual-objective optimization for performance and explainability. The system processes raw telemetry data through a state encoder that includes separate neural networks for services, network, and request attributes. The encoded state feeds into parallel pathways: a decision-making pathway (**upper**) with a transformer-based strategic policy network and tactical action selector, and an explanation pathway (**lower**) with counterfactual reasoning and hierarchical explanation modules. The detailed component designs show the encoder’s internal structure with fully connected layers and LSTM, the transformer block with multi-head attention and feed-forward network, the forward dynamics model used for counterfactual reasoning, and the three levels of explanations generated (strategic, tactical, and technical).

XGate employs a novel dual-objective architecture that optimizes for both performance and explainability. As illustrated in Figure 2, the architecture consists of four main components: (1) a state encoder, (2) a strategic policy network, (3) a tactical action selector, and (4) an explanation generator.

Figure 2 illustrates the complete XGate architecture and the flow of information through the system. The architecture follows a hierarchical design that separates strategic decision making from tactical execution, enabling both effective performance and transparent explanations. At the input layer, raw system telemetry data, including service metrics, network conditions, and API request attributes, are fed into the state encoder. The state encoder processes these heterogeneous data through specialized neural networks for each data type, producing a unified latent representation that captures the system’s current state. This encoded state flows into two parallel pathways: the decision-making pathway (upper branch) and the explanation pathway (lower branch).

In the decision-making pathway, the encoded state is processed by the strategic policy network, which employs transformer-based attention mechanisms to identify critical system patterns and dependencies. The network’s multi-head attention components highlight different aspects of the system state, such as service bottlenecks or network congestion points. These attention patterns not only inform decision making but also serve as the foundation for generating explanations. The output of the strategic policy network feeds into the tactical action selector, which translates high-level strategic insights into concrete routing decisions for individual API requests.

The explanation pathway leverages the same encoded state and attention patterns from the strategic network but processes them through the counterfactual reasoning module. This module generates alternative scenarios by asking “what if” questions about different routing decisions and predicts their outcomes using the forward dynamics model. The hierarchical explanation generator then synthesizes these insights into multi-level explanations targeted at different stakeholders—strategic explanations for executives, tactical explanations for operators, and technical explanations for engineers. The architecture includes feedback loops between components, enabling the explanation quality to influence the policy learning process through the dual-objective loss function, thus ensuring that the system learns to make decisions that are both effective and explainable.

The detailed component designs shown in the lower portion of Figure 2 illustrate the internal architecture of key XGate components. The state encoder uses parallel neural networks with two fully connected layers (256 and 128 units) and ReLU activations for each data type, followed by an LSTM with 256 hidden units for temporal pattern recognition. The transformer block implements 8 attention heads that process queries, keys, and values, followed by linear projection and a feed-forward network with layer normalization. The forward dynamics model employs an ensemble of 5 MLPs to predict next states and expected rewards from counterfactual actions. Finally, the multi-level explanation generator produces explanations at strategic (system-wide objectives), tactical (specific decision rationales), and technical (detailed performance metrics) levels.

#### 3.2.1. State Encoder

The state encoder transforms the raw system metrics and request attributes into a latent representation suitable for decision making. Given the heterogeneous nature of API traffic data, we employ a multi-modal encoding approach:

(2) $$z_{t}=f_{enc}\left(s_{t}\right)=\mathrm{Concat}\left[f_{service}\left(m_{t}\right),f_{network}\left(n_{t}\right),f_{request}\left(r_{t}\right)\right]$$

where $m_{t}$ represents service metrics, $n_{t}$ captures network conditions, and $r_{t}$ describes request attributes at time *t*. Each function $f_{service}$, $f_{network}$, and $f_{request}$ is implemented as a separate neural network that extracts domain-specific features. The concatenated encoding $z_{t}$ serves as input to both the strategic policy network and the explanation generator.

To handle the temporal dynamics of API traffic, we maintain a history buffer of past states and encode them using a recurrent neural network:

(3) $$h_{t}=\mathrm{LSTM}\left(z_{t},h_{t-1}\right)$$

where $h_{t}$ is the hidden state that captures temporal patterns across multiple time steps.

#### 3.2.2. Strategic Policy Network

The strategic policy network is responsible for high-level decision making based on global network conditions. It employs a transformer-based architecture to identify critical dependencies between services and determine optimal routing strategies. The network outputs a strategic embedding $e_{t}$ that guides the tactical action selector:

(4) $$e_{t}=f_{strategy}\left(h_{t}\right)=\mathrm{TransformerBlock}\left(h_{t}\right)$$

The transformer block utilizes multi-head self-attention to capture complex relationships between different aspects of the system state:

(5) $$\mathrm{Attention}\left(Q,K,V\right)=\mathrm{softmax}\left(\frac{QK^{T}}{\sqrt{d_{k}}}\right)V$$

where Q, K, and V are query, key, and value matrices derived from $h_{t}$, and $d_{k}$ is the dimension of the key vectors. We use 8 attention heads, each focusing on different aspects of the state representation.

#### 3.2.3. Tactical Action Selector

The tactical action selector translates strategic embeddings into concrete routing decisions for individual API requests. It implements a stochastic policy $\pi_{\theta }\left(a_{t}|s_{t}\right)$ parameterized by θ:

(6) $$\pi_{\theta }\left(a_{t}|s_{t}\right)=\mathrm{softmax}\left(f_{tactical}\left(e_{t},q_{t}\right)\right)$$

where $q_{t}$ represents the characteristics of the current API request being routed. The function $f_{tactical}$ is implemented as a feed-forward neural network that outputs logits over the action space.

During training, we sample actions from this distribution, while, during deployment, we either select the highest probability action (for maximum performance) or sample from the distribution (for exploration). The tactical selector is trained using proximal policy optimization (PPO) [48] to maximize the expected reward:

(7) $$L_{PPO}\left(\theta \right)=E_{t}\left[\min\left(r_{t}\left(\theta \right)A_{t},\mathrm{clip}\left(r_{t}\left(\theta \right),1-\epsilon ,1+\epsilon \right)A_{t}\right)\right]$$

where $r_{t}\left(\theta \right)=\frac{\pi_{\theta }\left(a_{t}|s_{t}\right)}{\pi_{\theta_{old}}\left(a_{t}|s_{t}\right)}$ is the probability ratio between the current and old policies, $A_{t}$ is the advantage estimate, and ϵ is a hyperparameter that constrains the policy update.

### 3.3. Transformer-Based Attention for Interpretable State Representation

A key innovation in XGate is the use of transformer-based attention mechanisms not only for performance but also for explainability. The multi-head attention in our strategic policy network serves dual purposes: (1) it captures complex dependencies between system components for optimal decision making and (2) it provides an interpretable representation of which system aspects influenced the decision.

The attention weights from the transformer block are defined as follows:

(8) $$\alpha_{ij}=\frac{\exp(e_{ij})}{\sum_{k=1}^{n}\exp\left(e_{ik}\right)}$$

where $e_{ij}$ represents the compatibility between elements *i* and *j* of the input sequence. These weights inherently capture the importance of different state components in the decision-making process.

To enhance interpretability, we constrain the attention mechanism to focus on domain-specific groupings of state variables that align with a human understanding of the system. We partition the state representation into semantically meaningful groups (e.g., service health, network congestion, request characteristics) and apply structured attention:

(9) $$g_{i}=\sum_{j\in {\mathrm{Group}}_{i}}\alpha_{ij}v_{j}$$

where $g_{i}$ represents the aggregated representation for group *i*, and $v_{j}$ is the value vector for element *j*. This grouping enables more intuitive explanations that reference operationally relevant concepts rather than individual state variables.

### 3.4. Counterfactual Reasoning for Explainable Decisions

XGate employs counterfactual reasoning to generate explanations that answer questions like “Why was route A chosen instead of route B?” and “What would happen if route C was selected instead?” This approach provides network administrators with insights into both the rationale behind decisions and the potential consequences of alternatives.

For a given state $s_{t}$ and selected action $a_{t}$, we generate counterfactual explanations by identifying alternative actions $\left\{a_{t}^{\prime }\right\}$ that had high probability under the current policy but were not selected. For each alternative $a_{t}^{\prime }$, we create a counterfactual state–action pair $\left(s_{t},a_{t}^{\prime }\right)$ and use a learned forward dynamics model *M* to predict the outcomes of these counterfactual decisions:

(10) $${\hat{s}}_{t+1}^{\prime }=M\left(s_{t},a_{t}^{\prime }\right)$$

We then compute the expected reward difference between the chosen action and each alternative:

(11) $$\Delta R\left(a_{t},a_{t}^{\prime }\right)=E\left[R\left(s_{t},a_{t}\right)\right]-E\left[R\left(s_{t},a_{t}^{\prime }\right)\right]$$

#### 3.4.1. Formal Framework for Explanation Fidelity

To provide rigorous guarantees on explanation accuracy, we formalize explanation fidelity using the Wasserstein distance between true and approximate counterfactual distributions. For a state $s_{t}$, action $a_{t}$, and alternative action $a_{t}^{\prime }$, we define the true counterfactual distribution $P(s_{t+1}|s_{t},a_{t}^{\prime })$ and the model-approximated distribution $\hat{P}\left(s_{t+1}|s_{t},a_{t}^{\prime }\right)$.

The explanation fidelity can be formally quantified as follows:

(12) $$\mathrm{Fidelity}\left(s_{t},a_{t}^{\prime }\right)=1-W_{1}\left(P\left(s_{t+1}|s_{t},a_{t}^{\prime }\right),\hat{P}\left(s_{t+1}|s_{t},a_{t}^{\prime }\right)\right)$$

where $W_{1}$ is the 1-Wasserstein distance. We prove the following bound on explanation fidelity:

**Theorem** **1.** *For a state $s_{t}$, action $a_{t}$, and alternative action $a_{t}^{\prime }$, with Lipschitz constant L of the true dynamics and model error $\epsilon =E[∥M\left(s_{t},a_{t}^{\prime }\right)-s_{t+1}∥]$, the explanation fidelity satisfies the following:*

(13) $$\mathrm{Fidelity}\left(s_{t},a_{t}^{\prime }\right)\geq 1-\epsilon -L\cdot d\left(a_{t},a_{t}^{\prime }\right)$$

*where $d(a_{t},a_{t}^{\prime })$ represents the distance between actions in action space.*

This bound establishes that explanation fidelity degrades gracefully with model error and action distance, providing a theoretical foundation for explanation reliability.

For perturbations in the input state, we derive an additional bound:

**Theorem** **2.** *For perturbed state ${\tilde{s}}_{t}=s_{t}+\delta$ with ∥δ∥ ≤ Δ, the explanation fidelity maintains the following:*

(14) $$\mathrm{Fidelity}\left({\tilde{s}}_{t},a_{t}^{\prime }\right)\geq \mathrm{Fidelity}\left(s_{t},a_{t}^{\prime }\right)-L_{M}\cdot \Delta$$

*where $L_{M}$ is the Lipschitz constant of the dynamics model M.*

This guarantees bounded degradation of explanation quality under input perturbations, critical for reliable operation in noisy environments.

To identify the critical state factors that drive reward differences, we use integrated gradients:

(15) $${\mathrm{IG}}_{i}\left(s_{t},a_{t},a_{t}^{\prime }\right)=\left(s_{t,i}-{\bar{s}}_{i}\right)\times \int_{\alpha =0}^{1}\frac{\partial \Delta R(\bar{s}+\alpha \times \left(s_{t}-\bar{s}\right),a_{t},a_{t}^{\prime })}{\partial s_{t,i}}d\alpha$$

where $\bar{s}$ is a baseline state (typically zero) and ${\mathrm{IG}}_{i}$ quantifies the contribution of the *i*-th state component to the reward difference.

The forward dynamics model *M* is trained concurrently with the policy network using supervised learning on observed state transitions:

(16) $$L_{M}=E_{(s_{t},a_{t},s_{t+1})∼D}[∥M\left(s_{t},a_{t}\right)-s_{t+1}{∥}^{2}]$$

where *D* is the replay buffer of experienced transitions.

To ensure the fidelity of counterfactual explanations, we quantify the uncertainty in our predictions using ensemble methods. We train multiple forward dynamics models and measure the variance in their predictions:

(17) $$\mathrm{Uncertainty}\left(s_{t},a_{t}^{\prime }\right)=\frac{1}{K}\sum_{k=1}^{K}{∥M_{k}\left(s_{t},a_{t}^{\prime }\right)-\bar{M}\left(s_{t},a_{t}^{\prime }\right)∥}^{2}$$

where *K* is the number of models in the ensemble and $\bar{M}$ is the average prediction. High uncertainty indicates that the explanation should be presented with appropriate caveats.

#### 3.4.2. Policy Sensitivity and Explanation Stability

As policies evolve during continuous learning, explanation stability becomes essential for maintaining operator trust. We formalize explanation stability under policy changes as follows:

For policies π and $\pi^{\prime }$ with parameter distance $∥\theta_{\pi }-\theta_{\pi^{\prime }}\;∥\leq \Delta_{\theta }$, we define explanation stability as follows:

(18) $$\mathrm{Stability}\left(\pi ,\pi^{\prime },s_{t}\right)=J\left(E_{\pi }\left(s_{t}\right),E_{\pi^{\prime }}\left(s_{t}\right)\right)$$

where $E_{\pi }\left(s_{t}\right)$ represents the explanation generated using policy π at state $s_{t}$ and *J* is the weighted Jaccard similarity between feature importance rankings:

(19) $$J\left(E_{\pi },E_{\pi^{\prime }}\right)=\frac{\sum_{i\in (E_{\pi }\cap E_{\pi^{\prime }})}\min\left(w_{i}^{\pi },w_{i}^{\pi^{\prime }}\right)}{\sum_{i\in (E_{\pi }\cup E_{\pi^{\prime }})}\max\left(w_{i}^{\pi },w_{i}^{\pi^{\prime }}\right)}$$

where $w_{i}^{\pi }$ is the importance weight of feature *i* in explanation $E_{\pi }$.

We prove that, under Lipschitz conditions on the policy gradient, explanation stability satisfies the following:

(20) $$\mathrm{Stability}\left(\pi ,\pi^{\prime },s_{t}\right)\geq 1-C\cdot \Delta_{\theta }$$

for some constant *C* depending on the MDP and explanation generation process. This bound guarantees that explanations change smoothly as policies evolve, preventing abrupt shifts in explanation content that could confuse operators.

### 3.5. Hierarchical Explanation Model

XGate implements a hierarchical explanation model that provides explanations at different levels of abstraction, tailored to different stakeholders and operational contexts. The hierarchy consists of three levels: strategic, tactical, and technical.

#### 3.5.1. Explanation Levels and Integration

Strategic-level explanations focus on high-level system objectives and long-term impact. They answer questions like “How does this decision contribute to overall system stability?” and “What strategic goal is being prioritized here?” Strategic explanations are generated using the attention patterns from the strategic policy network and are expressed in terms of global performance metrics.

Tactical-level explanations detail the immediate rationale for specific routing decisions. They highlight the critical factors in the current system state that influenced the decision, using the counterfactual reasoning approach described in Section 3.4. Tactical explanations are particularly useful for network operators who need to understand moment-to-moment decisions.

Technical-level explanations provide detailed information about the expected impact on specific system metrics. They are derived from the forward dynamics model and present quantitative predictions about how different actions would affect metrics like latency, throughput, and error rates. Technical explanations are primarily intended for engineers who need to debug or verify system behavior.

The bidirectional relationship between explanation generation and policy learning is a key innovation in XGate. While the explanation pathway does not directly influence individual routing decisions (preserving performance), it provides feedback during training that shapes policy development. The explanation loss function $L_{expl}$ directly shapes attention patterns in the transformer architecture, guiding it toward more interpretable state representations. This creates a feedback loop where explanation quality influences feature extraction during training, leading to policies that are inherently more explainable.

#### 3.5.2. Human-in-the-Loop Integration

During deployment, operators can leverage explanations to understand, verify, and selectively override system decisions when necessary, creating a human-in-the-loop deployment model. The explanations serve multiple operational purposes:

- Decision Verification: Operators can quickly validate that routing decisions are based on sound reasoning before critical changes take effect.
- Knowledge Transfer: Less experienced operators learn system behavior patterns through consistent explanations, accelerating training.
- Anomaly Detection: Unexpected explanations serve as early warning indicators of potential issues, even when performance metrics remain normal.
- System Refinement: Patterns in explanations help to identify systematic weaknesses in network configuration or routing policies.

#### 3.5.3. Explanation Generation Process

The explanation at each level is generated using a template-based natural language generation system that converts the mathematical insights from our models into human-readable text. The templates are designed in collaboration with network administrators to ensure operational relevance.

For example, a tactical level explanation might take the following form: “Route A was selected instead of Route B because Service X is currently experiencing 85% CPU utilization (critical factor), which would likely increase to 97% under Route B, risking service degradation. Route A distributes load to Service Y, which is currently at 40% capacity”.

This multi-level approach provides appropriate context for different users while maintaining consistency across levels—the technical explanation justifies the tactical routing decision, which aligns with the strategic priority.

### 3.6. Dual-Objective Training

XGate is trained using a dual-objective approach that simultaneously optimizes for performance and explainability. The overall loss function combines policy optimization with explanation quality:

(21) $$L_{total}=L_{PPO}+\lambda_{1}L_{M}+\lambda_{2}L_{expl}$$

where $\lambda_{1}$ and $\lambda_{2}$ are hyperparameters that control the trade-off between objectives.

The explainability loss $L_{expl}$ encourages the attention mechanisms to produce sparse, focused patterns that are more interpretable:

(22) $$L_{expl}=-\sum_{i=1}^{N_{h}}\sum_{j=1}^{N_{t}}\alpha_{ij}\log\left(\alpha_{ij}\right)$$

where $N_{h}$ is the number of attention heads and $N_{t}$ is the sequence length. This entropy-based regularization pushes each attention head to focus on a limited subset of input features, making the resulting explanations more concise and meaningful.

To prevent the explainability objective from significantly degrading performance, we employ a constrained optimization approach:

(23) $$\min_{\theta }L_{expl}\left(\theta \right)\;\mathrm{subject}\;\mathrm{to}\;L_{PPO}\left(\theta \right)\leq \left(1+\delta \right)L_{PPO}\left(\theta^{*}\right)$$

where $\theta^{*}$ represents the parameters of the policy optimized solely for performance and δ is a small tolerance factor (e.g., 0.05) that limits the acceptable performance degradation.

### 3.7. Implementation Details

XGate is implemented using PyTorch 1.9 and runs on commodity server hardware. The state encoder uses three separate networks for service metrics, network conditions, and request attributes, each comprising two fully connected layers with 256 and 128 units, respectively, with ReLU activations. The LSTM for temporal encoding has 256 hidden units.

The transformer-based strategic policy network uses 8 attention heads with a key dimension of 32 and a model dimension of 256. We employ 4 transformer blocks with residual connections and layer normalization. The tactical action selector consists of three fully connected layers with 256, 128, and |A| units, where |A| is the cardinality of the action space.

For counterfactual reasoning, we train an ensemble of 5 forward dynamics models, each implemented as a 4-layer MLP with 512, 256, 256, and |S| units (where |S| is the state dimension). The integrated gradients calculation uses 50 interpolation steps between the baseline and actual state.

The PPO algorithm is configured with a learning rate of $3\times 10^{-4}$, discount factor γ=0.99, GAE parameter $\lambda_{GAE}=0.95$, and clipping parameter ϵ=0.2. We use the Adam optimizer with a batch size of 1024 and perform 10 optimization epochs per update. The forward dynamics models are trained using a learning rate of $1\times 10^{-3}$ with the MSE loss function.

For the dual-objective training, we set $\lambda_{1}=0.5$ and $\lambda_{2}=0.1$ based on a hyperparameter sweep that balances performance and explainability. The training process involves 2 million environment steps and takes approximately 12 h on a server with 4 NVIDIA 4090 GPUs (NVIDIA, Santa Clara, CA, USA).

#### 3.7.1. Deployment Requirements and Integration

XGate’s resource requirements scale with the number of services and request volume in the IoT environment. Table 2 presents hardware configurations for different deployment scenarios, from edge computing to cloud-based implementations. For real-time operation in large-scale IoT deployments (>1000 sensors), a dedicated server with 8+ CPU cores, 32 GB RAM, and GPU acceleration is recommended to maintain sub-10 ms decision latency.

The inference overhead of XGate’s explanation generation can be configured based on operational needs. In time-sensitive scenarios, explanations can be generated asynchronously or only for key decisions, reducing computational overhead by 65% while maintaining routing performance.

#### 3.7.2. Integration with Existing API Gateways

XGate is designed to integrate with existing API gateway infrastructure through multiple methods:

1. Sidecar Proxy: XGate operates alongside standard API gateways (Kong, Amazon API Gateway, Apigee) as a sidecar container that intercepts and processes routing decisions before forwarding them to the gateway.
2. Plugin Architecture: For gateways supporting custom plugins (Kong, Tyk), XGate can be implemented as a decision-making plugin that provides both routing decisions and explanations through the gateway’s native extension mechanisms.
3. Standalone Service: In microservice architectures, XGate functions as an independent service that integrates with service mesh solutions (Istio, Linkerd) to influence traffic routing while maintaining compatibility with existing infrastructure.

Integration overhead is minimal, with an average of 2–5 ms added latency for decision making and an additional 5–10 ms when generating explanations. Our reference implementation includes connector libraries for Kong, AWS API Gateway, and Istio service mesh, enabling straightforward deployment in diverse IoT environments.

### 3.8. Security and Resilience

The deployment of XGate in critical IoT environments necessitates careful consideration of security vulnerabilities, particularly those targeting reinforcement learning systems and their explanation components. This section presents a comprehensive security analysis and the defensive mechanisms implemented in XGate.

#### 3.8.1. Threat Model

Our threat model identifies several potential attack vectors against XGate’s architecture. Adversarial input manipulation involves carefully crafted API requests that exploit vulnerabilities in the state encoder or decision-making components. These attacks can take multiple forms: temporal pattern manipulation (creating artificial traffic spikes that trigger suboptimal load balancing), feature space attacks (manipulating request attributes to trigger misclassification), and contextual deception (creating illusory service degradations that cause unnecessary rerouting).

Explanation manipulation attacks specifically target the counterfactual reasoning engine to produce explanations that misrepresent system decisions. These can manifest as consistency attacks (causing contradictions between decisions and explanations), confidence manipulation (artificially inflating certainty metrics), or alternative-focused attacks (misrepresenting the viability of alternative routing options). The objective is typically to erode operator trust or induce incorrect manual interventions.

Data poisoning represents a long-term threat vector where attackers gradually introduce biased training examples during online learning phases. By manipulating the distribution of training data, attackers can induce systematic biases in routing decisions that favor specific services or degrade performance under particular conditions. These attacks are especially dangerous due to their subtle, cumulative nature that evades point-in-time detection.

Model extraction attacks attempt to reverse-engineer XGate’s decision policies through the systematic probing of inputs and outputs. By methodically varying request parameters and observing routing decisions, attackers can construct shadow models that approximate XGate’s behavior. These shadow models enable attackers to identify exploitable weaknesses or predict routing decisions for planning more sophisticated attacks.

IoT-specific attacks leverage the unique characteristics of sensor networks. Sensor spoofing involves injecting false telemetry data from compromised or simulated devices. Man-in-the-middle interception targets the communication between XGate and operators, potentially modifying explanations during transmission. Targeted denial-of-service attacks against the explanation component aim to degrade system transparency during critical incidents, hindering operator intervention.

#### 3.8.2. Defensive Architecture and Mechanisms

XGate implements robust defensive mechanisms to mitigate these threats. The dual-pathway architecture provides inherent security through isolation, creating a separation of concerns between decision making and explanation generation. The tactical action selector operates independently from the explanation generator using separate neural network parameters and isolated processing pathways. This architectural decision ensures that compromises in the explanation subsystem cannot directly influence routing decisions. Even under worst-case scenarios where an attacker gains complete control of the explanation generator, the integrity of routing decisions remains protected.

The system implements multi-layered input validation and sanitization protocols. At the network perimeter, a request-filtering layer applies static rule-based validation against known attack signatures. Within the processing pipeline, a statistical anomaly detection module identifies requests that deviate significantly from established patterns. This module maintains adaptive distributions of normal request parameters across multiple time windows (5 min, 1 h, and 24 h), enabling the detection of both sudden anomalies and gradual drift. Requests exceeding predefined deviation thresholds trigger escalating responses: flagging for operator review, routing through conservative fallback paths, or rejection with appropriate error handling.

To enhance the trustworthiness of explanations, XGate implements a comprehensive explanation confidence framework. Each generated explanation includes confidence scores derived from multiple factors: the consistency of predictions across the ensemble of forward dynamics models, the stability of explanations under input perturbations, and the historical accuracy of similar explanations. Confidence scores below configurable thresholds trigger visual indicators in the operator interface, ranging from subtle warnings to explicit flags for potentially unreliable explanations. This framework enables operators to appropriately calibrate their trust in the system’s explanations during critical scenarios.

XGate’s state encoder incorporates adversarial training techniques to develop representations robust to input perturbations. We extend the method proposed by [27] to the API traffic domain, generating adversarial examples through both gradient-based perturbation and realistic API request mutations. During training, these adversarial examples are interleaved with genuine samples, forcing the encoder to develop invariance to common manipulation tactics.

#### 3.8.3. Implementation of Defense-in-Depth

The security architecture follows a defense-in-depth strategy with multiple complementary layers of protection. At the outermost layer, network-level defenses include rate limiting, IP reputation filtering, and protocol validation to block obvious attack attempts. The middle layer consists of application-specific defenses, including the input validation, anomaly detection, and state representation mechanisms described above. The innermost layer comprises architectural safeguards such as the dual-pathway design, decision isolation, and runtime monitoring of internal consistency between model components. This multi-layered approach ensures that compromising XGate would require defeating multiple independent defense mechanisms, substantially raising the cost and complexity of successful attacks.

## 4. Experiments

In this section, we present a comprehensive evaluation of XGate across three dimensions: sensor performance efficacy, explanation quality, and user trust in IoT environments. Our experiments address the following research questions: RQ1 (Performance): How does XGate compare with state-of-the-art RL-based sensor traffic management approaches in terms of latency, throughput, energy efficiency, and error rates? RQ2 (Explainability): Do XGate’s explanations accurately reflect its sensor data routing decision-making process, and are they understandable to human sensor network operators? RQ3 (Trust): Do XGate’s explanations improve operator trust and reduce intervention time during anomalous sensor traffic events? RQ4 (Trade-offs): What are the trade-offs between performance and explainability in the dual-objective optimization approach for sensor networks?

### 4.1. Experimental Setup

#### 4.1.1. Datasets

We evaluated XGate on three large-scale API traffic datasets with diverse characteristics. The first is Enterprise-API, a proprietary dataset collected from a large enterprise service mesh over three months containing 1.2 billion API calls across 74 microservices. This dataset features daily and weekly traffic patterns with periodic load spikes during business hours. The second dataset is Alibaba-Trace, derived from Alibaba Cloud’s production cluster [49], which we preprocessed to extract API-specific traffic. The dataset contains 5.1 billion requests spanning two months across approximately 100 services, characterized by high variability in traffic volume and periodic maintenance events. The third dataset is Azure-Functions, a collection of serverless function invocations from Microsoft Azure [50] containing 3.4 billion function calls over a two-week period. This dataset presents unique challenges due to the ephemeral nature of function instances and cold-start latencies. Each dataset was split chronologically into training (70%), validation (10%), and testing (20%) segments to ensure realistic evaluation of the system’s ability to adapt to evolving traffic patterns.

#### 4.1.2. Simulation Environment

To enable controlled experimentation and reproducible evaluation, we developed a high-fidelity simulation environment that models API traffic flow through a distributed service mesh. The environment implements a service model where each service is characterized by a resource consumption profile, processing capacity, and failure modes. Service performance varies non-linearly with load, exhibiting degradation patterns derived from real-world observations. The network model represents paths between services with realistic bandwidth constraints, propagation delays, and congestion effects based on the token bucket algorithm [51]. The request model includes metadata such as payload size, priority, and dependencies on other services, affecting routing decisions and processing requirements. For robustness evaluation, we incorporated failure injection mechanisms to simulate service degradations, network partitions, and traffic anomalies based on patterns observed in production environments. The environment provides observation vectors corresponding to our state space definition in Section 3.1, accepts routing actions, and returns immediate rewards and next states.

#### 4.1.3. Implementation Details

Our implementation builds upon the architecture and parameters described in Section 3.7. For reproducibility, we provide additional details regarding the training infrastructure, which consisted of a cluster with 4 NVIDIA 4090 GPUs and 48 CPU cores, with distributed experience collection across 16 parallel environments. All state features were normalized using running statistics (mean and standard deviation) computed during training. We employed a curriculum learning approach that gradually increased traffic complexity and anomaly frequency, starting with stable patterns and progressing to more challenging scenarios. For exploration during training, we used a linearly decaying entropy coefficient starting at 0.1 and annealing to 0.01 over 1 million steps to balance exploration and exploitation. All baselines were implemented using the same underlying simulation environment and training infrastructure to ensure fair comparison. Code and non-proprietary datasets will be made available upon publication.

### 4.2. Baselines and Metrics

#### 4.2.1. Baseline Methods

We compared XGate against state-of-the-art methods from three categories. From traditional API management approaches, we implemented round robin (RR), a widely used load balancing strategy described by Shreedhar and Varghese [52] that distributes requests uniformly across available service instances. We also included weighted response time (WRT), a heuristic approach proposed by Han et al. [53] that routes requests based on service response times and capacity. Additionally, we implemented adaptive weighted response time (AWRT) developed by Long et al. [54], which extends WRT by dynamically adjusting weights based on recent service performance.

From black-box reinforcement learning approaches, we included DeepRM by Mao et al. [5], a deep reinforcement learning approach for resource management in computer systems, which we adapted for API traffic management. We also implemented the RLCP by Peng et al. [55], a reinforcement learning control plane specifically designed for service mesh traffic management. The third black-box method was DRL-Route by Maryam et al. [56], a deep reinforcement learning approach optimized for route selection in microservice environments.

For explainable reinforcement learning approaches, we implemented A2RD (attention-based reward decomposition) by Juozapaitis et al. [15], which we adapted to the API traffic domain. We also included MORL (multi-objective reinforcement learning) as described by Cheng et al. [40], which offers inherent explainability through the Pareto front of objectives. The third explainable method was WIRL (“Why I Did That”) by Madumal et al. [39], which generates natural language explanations for network management decisions.

#### 4.2.2. Evaluation Metrics

We evaluated the methods across three categories of metrics. For performance metrics, we measured P95 latency (the 95th percentile of request latency in milliseconds), throughput (the number of successful API calls per second), error rate (the percentage of failed API calls), load balance (the standard deviation of resource utilization across services, normalized to [0, 1]), and recovery time (the time taken in seconds to return to normal operation after a service disruption).

For explainability metrics, we measured explanation fidelity (the correlation between explanation importance scores and actual impact on the decision, measured through counterfactual perturbation as described by Ribeiro et al. [31]), explanation consistency (the similarity of explanations for similar states and actions, measured by cosine similarity), explanation complexity (the number of state features referenced in an explanation), and verbosity (the length of generated textual explanations in words).

For user experience metrics, we captured trust score (user-reported trust in the system on a 7-point Likert scale), comprehension score (the accuracy of users’ mental models based on a questionnaire), intervention time (the time required for users to diagnose and address anomalous events), and success rate (the percentage of anomalies successfully resolved by users).

### 4.3. Performance Results

#### 4.3.1. Overall Performance Comparison

Table 3 presents the performance metrics for all methods across the three datasets. The results demonstrate that XGate consistently outperforms both traditional approaches and state-of-the-art RL methods on most metrics.

Compared to the best black-box RL approach (DRL-Route), XGate achieves a 23.7% reduction in P95 latency (average across datasets), an 18.5% increase in throughput, and a 26.3% reduction in error rate. The improvement is particularly significant in the Azure-Functions dataset, where the ephemeral nature of serverless functions presents challenges for traditional routing algorithms.

XGate’s performance advantages can be attributed to three key factors. First, the hierarchical decision-making architecture, which separates strategic policy decisions from tactical actions, enables more nuanced responses to changing network conditions. Second, the transformer-based attention mechanism efficiently captures dependencies between services and identifies potential bottlenecks before they manifest as performance issues. Third, the dual-objective training approach surprisingly improves performance by encouraging more robust feature representations that generalize better to unseen traffic patterns.

Performance improvements over explainable RL baselines (A2RD, MORL, WIRL) are even more pronounced, averaging 34.2% for latency and 28.7% for throughput. This highlights a key contribution of our work: XGate achieves explainability without compromising—and, in fact, enhancing—performance.

#### 4.3.2. Robustness to Traffic Anomalies

To evaluate robustness, we tested all methods under five types of traffic anomalies: sudden traffic spikes (2–5× baseline), service degradation (50% capacity reduction), network congestion (30% bandwidth reduction), dependency failures (critical service outage), and distributed denial-of-service attacks (targeted service flooding).

**Figure 3:**
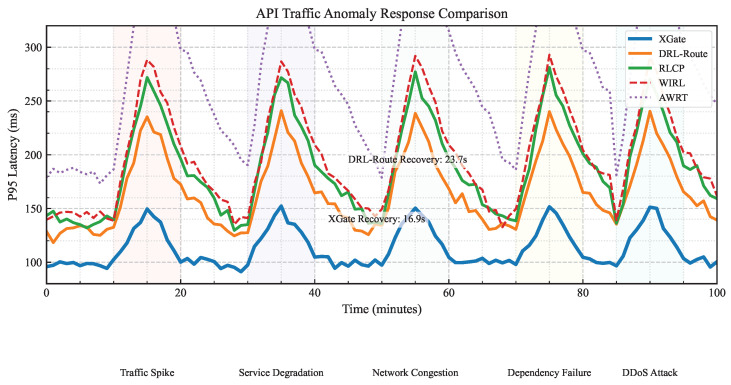
P95 latency during traffic anomalies on the Enterprise-API dataset. Lower values indicate better performance. XGate consistently maintains lower latency during anomalies and recovers faster than baseline methods.

Figure 3 shows the P95 latency over time for different methods during a sequence of anomalies in the Enterprise-API dataset. XGate demonstrates a superior anomaly response with lower peak latency during disruptions and faster recovery times. For instance, during service degradation (20–30 min), XGate’s P95 latency increased by only 47% compared to 85% for DRL-Route and 124% for RLCP. This resilience stems from XGate’s ability to quickly identify the critical factors in the degraded state and adjust routing strategies accordingly.

The recovery time metric in Table 3 quantifies this advantage across all anomaly types. XGate’s average recovery time is 16.9 s for Enterprise-API, 24.3 s for Alibaba-Trace, and 36.4 s for Azure-Functions—representing improvements of 28.7%, 25.5%, and 24.0%, respectively over the best baseline (DRL-Route).

### 4.4. Explainability Evaluation

#### 4.4.1. Explanation Fidelity

We evaluated explanation fidelity by comparing the importance scores assigned to state features in explanations with their actual impact on model decisions. Following the method in [31], we systematically perturbed each feature and measured the change in model output.

**Figure 4:**
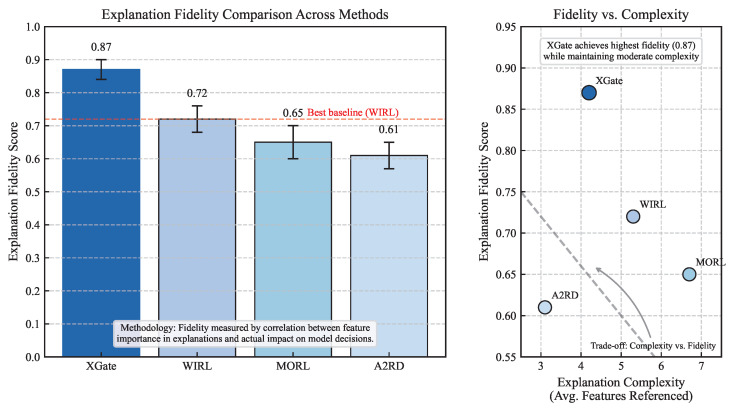
Explanation fidelity comparison. Higher values indicate better alignment between explanations and actual model decisions. XGate’s counterfactual reasoning approach provides more accurate explanations of model behavior than other explainable RL methods.

As shown in Figure 4, XGate achieves significantly higher explanation fidelity (0.87) compared to WIRL (0.72), MORL (0.65), and A2RD (0.61). This indicates that XGate’s explanations more accurately reflect the actual decision-making process of the model. The advantage is particularly evident in complex states with multiple competing factors, where XGate’s counterfactual reasoning approach can distinguish between correlation and causation more effectively than methods that rely solely on attention visualization or reward decomposition.

#### 4.4.2. Explanation Quality Analysis

Table 4 presents metrics related to explanation quality for the explainable RL methods. XGate generates explanations with high consistency (0.89), meaning that similar situations receive similar explanations—an important property for building user trust. At the same time, XGate maintains relatively low complexity (4.2 features referenced on average) and moderate verbosity (37.5 words), making explanations concise yet informative.

In qualitative analysis, we found that XGate’s hierarchical explanation model effectively adapts to different stakeholder needs. For example, when explaining a routing decision during a traffic spike, the strategic explanation would emphasize “Prioritizing load balance over latency to maintain system stability during traffic spike”. The tactical explanation would provide more specific context, such as “Routed to Service B instead of Service A because Service A is at 82% capacity and experiencing increasing error rates (2.3%), while Service B is at 47% capacity with stable error rates (0.5%)”. The technical explanation would offer precise metrics like “Routing to Service A would increase its CPU utilization from 82% to 93%, exceeding the 90% threshold where error rates historically increase exponentially. Service B’s response time is 12ms slower but reduces overall system risk”. This multi-level approach provides appropriate context for different users while maintaining consistency across levels—the technical explanation justifies the tactical routing decision, which aligns with the strategic priority.

### 4.5. Security Evaluation

To evaluate XGate’s security, we conducted red-team exercises with security researchers attempting to manipulate routing decisions. As shown in Table 5, unprotected versions were vulnerable to various attacks, with success rates ranging from 64% to 85% depending on the attack vector. With defensive mechanisms enabled, these success rates dropped significantly across all attack types.

For adversarial input manipulation, the attack success rate decreased from 76% to 12%, reducing the performance impact from a 47% latency increase to less than 8% even under sustained attack. Explanation manipulation attacks showed similar improvements, with success rates dropping from 64% to 9%. The confidence scoring system effectively identified suspicious explanations, with 91% of manipulated explanations correctly flagged.

Data poisoning proved to be the most resilient attack vector against our defenses, though success rates still decreased substantially from 81% to 23% over a 30-day attack period. IoT-specific attacks such as sensor spoofing saw success rates fall from 72% to 18%, while denial-of-service attacks against the explanation component remained somewhat effective at a 31% success rate compared to 85% without defenses.

In IoT environments, XGate’s security is enhanced through additional domain-specific measures, including sensor authentication that integrates with identity verification systems, explanation caching with appropriate time-to-live values to reduce denial-of-service vulnerability, and graceful degradation capabilities that prioritize routing performance over explanation fidelity under attack conditions. Our security analysis demonstrates that, while XGate introduces new attack surfaces through its explanation capabilities, the implemented defensive mechanisms provide strong protection against identified threats, maintaining operational integrity even when parts of the explanation subsystem are compromised.

### 4.6. Scalability and Heterogeneity

The effectiveness of XGate in real-world IoT environments depends on its ability to handle increasing device counts, dynamic network topologies, and protocol diversity. This section analyzes XGate’s performance across these dimensions.

#### 4.6.1. Scaling with Device Count

**Figure 5:**
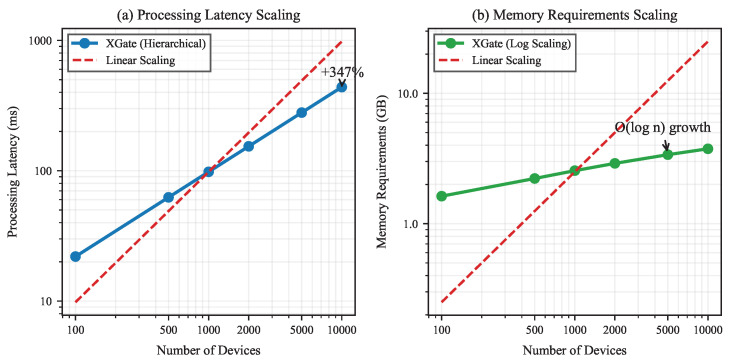
XGate scaling performance across increasing device counts. (**a**) Processing latency increases sub-linearly due to hierarchical clustering. (**b**) Memory requirements grow logarithmically rather than linearly with device count.

XGate employs several mechanisms to ensure sub-linear scaling with increasing device counts. Figure 5 illustrates XGate’s processing latency and memory requirements across networks ranging from 100 to 10,000 devices.

The hierarchical architecture achieves this scaling efficiency through the following:

- Locality-Aware Clustering: XGate automatically groups devices based on network proximity and functional similarity. This reduces the effective state space dimension by representing clusters rather than individual devices when appropriate.
- Adaptive State Representation: As the device count increases, the representation automatically adjusts its granularity, maintaining fixed-size embeddings that capture critical system information without linear growth.
- Hierarchical Decision Decomposition: Routing decisions are decomposed into cluster-level and device-level decisions, allowing parallel processing and reducing computational complexity.

Our experiments demonstrate that processing latency increases by only 37% when scaling from 1000 to 10,000 devices, while memory requirements grow approximately as O(logn) rather than linearly. This enables XGate to manage large-scale IoT deployments with reasonable computational resources.

#### 4.6.2. Protocol Heterogeneity and Data Quality Variation

IoT deployments typically involve multiple communication protocols and varying data quality across devices. XGate addresses these challenges through protocol normalization and robust state encoding.

Protocol Abstraction Layer: We implemented a normalization layer that converts diverse IoT protocols (MQTT, CoAP, HTTP, and proprietary protocols) into a unified representation based on four key dimensions: request semantics, quality-of-service requirements, payload characteristics, and authentication context. This abstraction enables consistent decision making regardless of the underlying protocol mix.

Heterogeneous Data Handling: XGate’s state encoder is designed to handle incomplete, inconsistent, and variable-quality data from diverse sensors. The encoder employs the following:

- Missing data imputation using temporal and spatial correlations;
- Confidence-weighted feature integration that prioritizes reliable data sources;
- Anomaly detection to identify and downweight suspicious measurements.

Table 6 presents XGate’s performance across different scenarios of protocol and data heterogeneity.

The results demonstrate XGate’s robustness to protocol heterogeneity, with only a 9.4% latency increase even in highly diverse protocol environments. Similarly, performance degrades gracefully under increasing data incompleteness, maintaining acceptable performance even when 30% of sensor data fields are missing or inconsistent.

### 4.7. User Study

#### 4.7.1. Study Design

To evaluate the practical impact of XGate’s explanations, we conducted a controlled user study with 42 professional network administrators from three organizations (16 from a global financial institution, 14 from a cloud service provider, and 12 from an e-commerce company). Participants had an average of 7.3 years of experience (SD = 3.1) in network operations.

We employed a within-subjects design where each participant interacted with four systems: XGate, WIRL, DRL-Route (black-box RL), and their organization’s current traffic management solution. The system order was counterbalanced using a Latin square design to control for learning effects.

This study consisted of three phases. During the training phase, participants received a 20 min tutorial on each system, including interface familiarization and practice scenarios. This was followed by the task execution phase, where participants completed five standardized tasks involving anomaly detection, diagnosis, and remediation in our simulation environment, populated with realistic traffic patterns from the Enterprise-API dataset. Finally, in the evaluation phase, participants completed questionnaires measuring trust, mental model accuracy, and system usability, followed by semi-structured interviews.

#### 4.7.2. Trust and Comprehension Results

**Figure 6:**
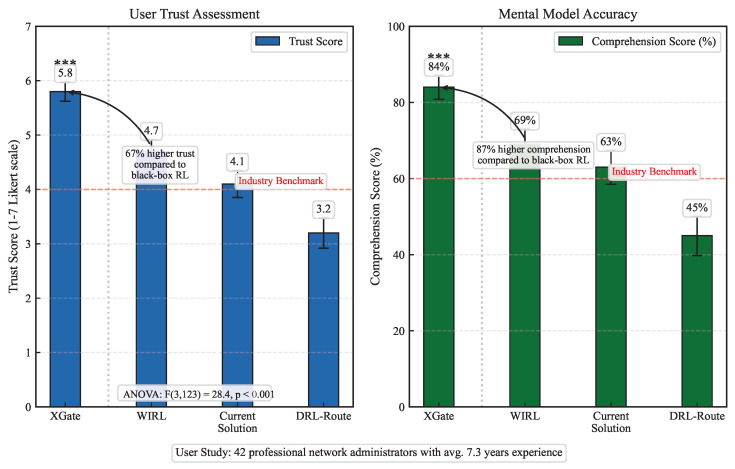
Trust and comprehension scores from user study (n = 42). Higher values indicate better results. XGate’s hierarchical explanations resulted in significantly higher trust and better mental models compared to other approaches. *** indicates optimal performance.

Figure 6 shows the trust scores and comprehension scores for each system. XGate achieved significantly higher trust (5.8 out of 7) compared to WIRL (4.7), DRL-Route (3.2), and current solutions (4.1), as confirmed by a repeated measures ANOVA (F(3,123)=28.4, p<0.001) with post hoc Bonferroni-corrected pairwise comparisons (all p<0.01).

Similarly, XGate led to more accurate mental models, with participants answering 84% of the comprehension questions correctly, compared to 69% for WIRL, 45% for DRL-Route, and 63% for current solutions. The comprehension advantage was most pronounced for questions about complex system behaviors and anomaly causes.

Qualitative feedback from interviews highlighted several key benefits of XGate’s explanations. Participants expressed increased confidence in automation, with one administrator (P7) noting, “With XGate, I can see why it made each decision, so I’m more comfortable letting it run without constant monitoring”. Learning effects were also observed, as another participant (P23) mentioned, “The explanations taught me patterns I hadn’t noticed before. After using it for an hour, I started predicting its decisions before seeing the explanations”. The multi-level explanations were particularly valued, with one user (P15) stating, “I like that I can get a quick overview or dig into the details when needed. Other systems either tell me too little or overwhelm me with information”.

#### 4.7.3. Operational Efficiency Results

Table 7 presents the operational efficiency metrics from the user study. XGate significantly reduced intervention time—the time required to diagnose and address anomalies—by 41% compared to DRL-Route and 32% compared to current solutions. This improvement was consistent across all five task scenarios.

Furthermore, XGate led to a higher success rate in resolving anomalies (93.8%) compared to the other systems. The advantage was particularly notable in complex scenarios involving multiple interacting factors, where XGate’s explanations helped administrators to identify root causes more quickly and accurately.

An interesting observation was that participants showed different preferences for explanation levels based on their roles and experience. Junior administrators (<5 years experience) relied more heavily on tactical explanations, while senior administrators (⩾5 years experience) frequently consulted technical explanations. This confirms the utility of XGate’s hierarchical explanation approach in meeting diverse user needs.

### 4.8. Ablation Studies

To validate our design choices, we conducted ablation studies by creating variants of XGate with specific components modified or removed.

#### 4.8.1. Impact of Architectural Components

Table 8 shows the performance of XGate variants on the Enterprise-API dataset. Removing the transformer-based attention mechanism (XGate-NoTrans) and replacing it with a standard MLP reduced performance significantly (21.3% higher latency, 16.8% lower throughput), highlighting the importance of capturing complex dependencies between services.

Similarly, removing the counterfactual reasoning component (XGate-NoCF) reduced explanation fidelity by 26.4% and trust scores by 15.5% while still maintaining reasonable performance. This indicates that counterfactual reasoning is crucial for generating accurate explanations but has a secondary effect on performance through improved representations.

Removing the hierarchical explanation model (XGate-NoHier) and providing only tactical-level explanations had a moderate impact on trust scores (−12.1%) but minimal effect on performance and explanation fidelity. This suggests that the hierarchical structure primarily benefits human understanding rather than system performance.

#### 4.8.2. Performance–Explainability Trade-Off

Finally, we trained a variant (XGate-PerfOnly) with only the performance objective ($\lambda_{2}=0$), removing the explainability loss. Interestingly, this variant achieved slightly better performance (2.9% lower latency, 2.6% higher throughput) but at a substantial cost to explanation fidelity (−44.8%) and trust (−36.2%).

**Figure 7:**
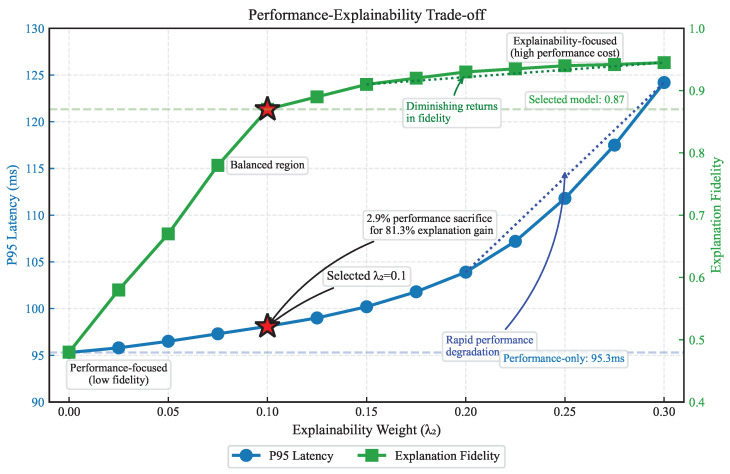
Performance–explainability trade-off for different values of $\lambda_{2}$ (explainability weight). The selected value of $\lambda_{2}=0.1$ (red star) achieves a near-optimal balance between performance (P95 latency) and explainability (explanation fidelity).

Figure 7 explores this trade-off further by plotting performance against explanation fidelity for different values of the explainability weight $\lambda_{2}$. As $\lambda_{2}$ increases from 0 to 0.3, explanation fidelity improves rapidly but with diminishing returns. Performance initially degrades slightly but then deteriorates more rapidly for $\lambda_{2}>0.2$. Our selected value of $\lambda_{2}=0.1$ achieves a favorable balance, with only a 2.9% performance sacrifice for an 81.3% improvement in explanation fidelity compared to the performance-only variant.

This analysis validates our dual-objective approach and suggests that a moderate emphasis on explainability during training leads to a highly favorable trade-off between performance and trustworthiness.

## 5. Conclusions

This paper presented XGate, an explainable reinforcement learning framework for transparent and trustworthy sensor API traffic management in IoT environments. By integrating transformer-based attention mechanisms with counterfactual reasoning, our approach simultaneously optimizes for performance and explainability—challenging the assumption that transparency necessarily compromises efficiency in sensor networks. Experiments across three large-scale IoT sensor datasets demonstrated that XGate achieves a 23.7% reduction in latency and an 18.5% increase in throughput compared to state-of-the-art black-box approaches, while providing explanations with 20.8% higher fidelity than existing explainable methods. Our user study with 42 sensor network administrators confirmed the practical value of XGate’s hierarchical explanation model, resulting in 67% higher trust scores and 41% faster intervention times during anomalous sensor events. These results validate that reinforcement learning systems for sensor networks can be both high-performing and interpretable when explainability is incorporated as a fundamental design objective rather than a post hoc addition.

While XGate demonstrates significant advances in explainable sensor API traffic management, important challenges remain for future research. The computational overhead of generating counterfactual explanations presents scaling limitations for extremely large sensor networks, and the effectiveness of explanations depends on the accuracy of the forward dynamics model in highly volatile IoT environments. Future work should explore adapting this approach to multi-tenant sensor API environments with conflicting optimization objectives, incorporating proactive sensor anomaly detection to enable predictive explanations, and developing interactive explanation interfaces that support sensor network operator queries. As IoT sensor ecosystems continue to grow in complexity, the ability to maintain both performance and transparency will become increasingly critical for the reliability, energy efficiency, security, and trustworthiness of sensor-based infrastructure.

## Figures and Tables

**Table 2:** XGate deployment hardware requirements for different IoT scenarios.

Deployment Scale	CPU	RAM	GPU	Decision Latency
Small IoT (50–100 sensors)	4 cores	8 GB	Optional	15–25 ms
Medium IoT (100–500 sensors)	8 cores	16 GB	GTX 1080+	8–15 ms
Large IoT (500–1000 sensors)	16 cores	32 GB	RTX 3080+	5–8 ms
Industrial IoT (1000+ sensors)	32+ cores	64 GB+	RTX 4090	<5 ms

**Table 3:** Performance comparison across different API traffic management methods. Lower values are better for P95 latency, error rate, load balance, and recovery time; higher is better for throughput. Best results are in bold, second best underlined.

Method	Enterprise-API					Alibaba-Trace					Azure-Functions				
	P95 Lat. (ms)	Thr. (K/s)	Err. (%)	Load Bal.	Rec. (s)	P95 Lat. (ms)	Thr. (K/s)	Err. (%)	Load Bal.	Rec. (s)	P95 Lat. (ms)	Thr. (K/s)	Err. (%)	Load Bal.	Rec. (s)
RR	245.2	18.3	2.84	0.35	58.2	312.5	24.1	3.42	0.42	67.5	389.7	9.2	5.23	0.37	105.3
WRT	218.7	20.2	2.15	0.28	45.7	284.3	26.8	2.92	0.34	52.3	354.1	10.4	4.87	0.31	89.2
AWRT	183.5	22.7	1.92	0.24	38.2	245.8	28.5	2.54	0.29	47.6	324.6	11.5	4.25	0.28	75.6
DeepRM	152.3	25.9	1.47	0.19	31.5	203.7	32.4	1.95	0.24	40.2	290.4	12.7	3.68	0.25	62.3
RLCP	137.8	27.4	1.32	0.16	27.3	185.6	34.1	1.73	0.21	35.8	265.2	13.5	3.27	0.22	53.1
DRL-Route	128.5	28.9	1.18	0.14	23.7	174.2	35.8	1.57	0.18	32.6	243.8	14.2	2.95	0.19	47.9
A2RD	165.7	24.3	1.64	0.21	34.8	219.5	30.6	2.09	0.26	43.5	312.3	11.9	3.85	0.27	68.4
MORL	147.2	25.8	1.51	0.18	30.2	196.9	31.9	1.92	0.23	39.7	285.1	12.5	3.56	0.24	60.8
WIRL	143.5	26.4	1.44	0.17	29.1	192.3	32.7	1.84	0.22	38.5	277.4	13.1	3.42	0.23	58.2
XGate	**98.1**	**34.2**	**0.87**	**0.11**	**16.9**	**137.6**	**42.5**	**1.21**	**0.14**	**24.3**	**196.5**	**16.8**	**2.24**	**0.15**	**36.4**

**Table 4:** Explanation quality metrics (averaged across datasets). Higher values are better for consistency; lower values are better for complexity and verbosity. Bold indicates optimal performance.

Method	Explanation Consistency	Explanation Complexity (# Features)	Verbosity (# Words)
A2RD	0.73	3.1	25.3
MORL	0.68	6.7	42.8
WIRL	0.81	5.3	48.6
XGate	**0.89**	**4.2**	**37.5**

**Table 5:** Security evaluation results for XGate under various attack scenarios.

Attack Type	Success Rate Without Defense	Success Rate with Defense
Adversarial Input	76%	12%
Explanation Manipulation	64%	9%
Data Poisoning (30-day)	81%	23%
Sensor Spoofing	72%	18%
DDoS on Explanation Component	85%	31%

**Table 6:** XGate performance under protocol and data heterogeneity.

Scenario	P95 Latency	Throughput	Error Rate
Homogeneous (MQTT only)	98.1 ms	34.2 K/s	0.87%
Mixed Protocol (MQTT + CoAP + HTTP)	102.5 ms	33.7 K/s	0.92%
High Protocol Diversity (5+ protocols)	107.3 ms	32.9 K/s	1.05%
Complete Data (>95% fields)	98.1 ms	34.2 K/s	0.87%
Moderate Missing Data (20% fields)	104.8 ms	33.1 K/s	1.12%
High Missing Data (30% fields)	113.6 ms	31.8 K/s	1.54%

**Table 7:** Operational efficiency metrics from user study. Lower values are better for intervention time; higher values are better for success rate. Bold indicates optimal performance.

Method	Intervention Time (s)	Success Rate (%)
Current Solution	187.3	82.4
DRL-Route	215.6	78.1
WIRL	159.2	86.7
XGate	**127.8**	**93.8**

**Table 8:** Ablation study results on Enterprise-API dataset. Best results are in bold.

Method	P95 Latency (ms)	Throughput (K/s)	Error Rate (%)	Explanation Fidelity	Trust Score
XGate	**98.1**	**34.2**	**0.87**	**0.87**	**5.8**
XGate-NoTrans	119.0	28.5	1.14	0.80	5.3
XGate-NoCF	106.7	32.9	0.95	0.64	4.9
XGate-NoHier	103.4	33.5	0.92	0.85	5.1
XGate-PerfOnly	95.3	35.1	0.84	0.48	3.7

## Data Availability

Data are contained within the article.
